# Effect of multiple pouring on the accuracy of casts made using 3D-printed custom trays with different spacer thicknesses: A research study

**DOI:** 10.34172/joddd.2020.005

**Published:** 2020

**Authors:** Sara Tavakolizadeh, Mohammad Javad Razaghi, Pedram Pakravan, Majid Sedaghat Monfared, Elaheh Beyabanaki, Rahab Ghoveizi

**Affiliations:** ^1^Department of Prosthodontics, Faculty of Dentistry, Shahid Beheshti University of Medical Sciences, Tehran, Iran; ^2^DDS, Shahid Beheshti Uiversity of Medical Sciences, Tehran, Iran

**Keywords:** Computer-aided design, dental impression, three-dimensional printing

## Abstract

***Background.*** This study aimed to evaluate the effect of different pouring times and spacer thicknesses on the three-dimensional accuracy of casts made of 3D-printed custom trays.

***Methods.*** A partial edentulous maxillary model was scanned for fabricating custom acrylic trays. Twenty custom trays were created using a CAD/CAM system and divided into two groups in terms of their spacer thicknesses (2 mm and 4 mm). All the trays were designed with 2-mm thickness, multiple retentive holes measuring 2 mm in diameter, and three interior seating stops (two on the edentulous ridge and one on the incisal edge of the central incisors). Impressions were made using monophasic polyvinyl siloxane and poured in two different times (one hour and 24 hours after removal) with type IV dental stone. All the casts were scanned to measure three distances (inter-buccal cusps, inter-palatal cusps, and inter-fossa distances) between the two first premolars. The data were analyzed with two-way ANOVA and Bonferroni test at a significance level of 0.05.

***Results.*** There was no significant difference between the 3D accuracy of casts made using two different spacer thicknesses poured at 1-hour and 24-hour intervals. However, there was a difference between casts made after 1 hour and 24 hours when using custom trays with 2 mm of spacer thickness in terms of inter-buccal distance.

***Conclusion.*** There was no significant difference between the accuracy of casts made using custom trays with either 2 or 4 mm of spacer thickness, which were poured 1 hour or 24 hours after tray removal.

## Introduction

An accurate and passive ﬁt of dental prostheses is necessary for the long-term health of tissues and longevity of the restoration. Achieving such fit partly depends on accurate recording of tissue surface during the impression-making procedure.^1^ In this regard, taking an accurate impression is very important to ensure the accuracy of the final restoration.

In this regard, the accuracy and dimensional stability of elastomeric impression materials, as one of the crucial factors of the impression procedure, has been studied.^2-4^ Monophasic polyvinyl siloxanes as an elastomeric impression material have been introduced for a one-step, single-mix impression technique for both syringe and tray loading. Therefore, these materials need to be used with custom trays to reduce dimensional changes.^2,3-5,6^ However, there is no agreement on the optimal thickness of impression material inside the tray (varying from 1 to 5 mm).^2,7-11^

Tray accuracy is also significant for creating a precise impression. Rigidity, fit, and provision of a sufficient uniform space for impression material are the most important features of a custom tray. Custom trays could be made using different materials and methods. The use of a tray spacer in custom trays has been advised to achieve the optimal thickness of impression materials in terms of accuracy and ﬂow, whereas the use of stock trays does not yield accurate results.^12-15^ One of the recent fabrication methods for customs trays is the use of 3D printing which offers advantages of being more accurate and less time-consuming as compared to conventional methods.^16^

This study aimed to evaluate and compare the effect of different spacer thicknesses for digitally made custom trays and also the pouring time of impressions made from monophasic polyvinyl siloxane on the accuracy of the resulting casts.

## Methods

**Figure 1:**
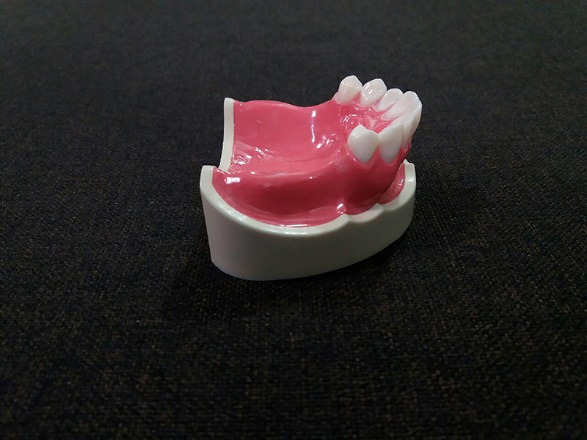
The original model of partially edentulous maxilla.

**Figure 2:**
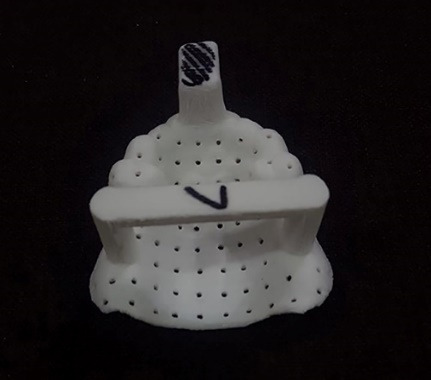
Digitally fabricated custom trays with 2- and 4-mm internal spaces.

**Figure 3:**
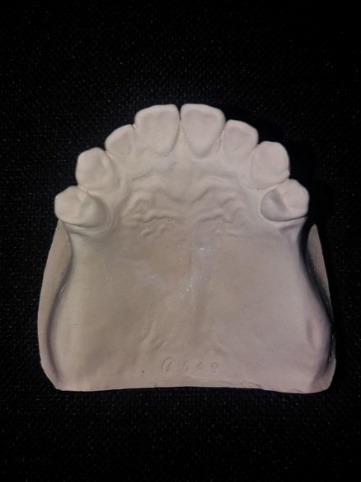
The final cast made using type IV dental stone.

**Figure 4:**
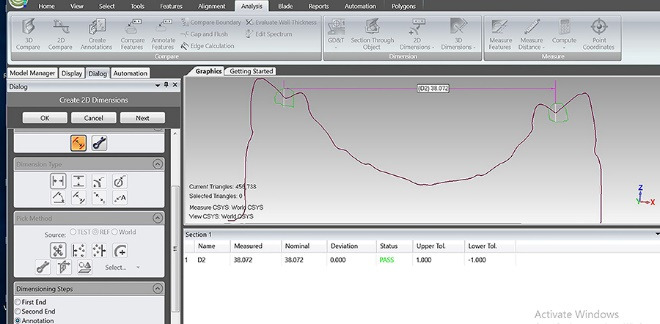
The measurement of inter-abutment distances.

A maxillary partial edentulous model (Nissin Dental Products INC, Japan) (Figure 1) was scanned by a three-dimensional scanner (Smart optic, GmbH, Germany) to create the model’s STL file. After transferring the digital file to a computer, mesh mixer software (Autodesk, Inc., USA) was used to design custom trays with 2-mm thickness and a handle (15 mm in length and height). Two groups were considered, one with 10 trays with 2-mm and the other group with 10 trays with 4-mm spacer thickness. In order to create mechanical retention, multiple 2-mm-diameter holes were made. Also, three seating stops were designed at the interior surface of trays at the distal end of the right and left edentulous areas and on the incisal edge of the central incisors. All the trays were made using a 3D printer (FDM em2, Tavana 3D, Iran) (Figure 2). The impressions were made using monophasic polyvinyl siloxane (Monopren transfer, Kettenbach GmbH & Co. KG) 15 minutes after using a tray adhesive (Monopren Transfer, Kettenbach GmbH & Co. KG) while applying a 3-kg pressure during its polymerization in a controlled-temperature environment (23±2°C) with a relative humidity of 50%±10%.^18^ The impressions were removed minutes later according to the impression material’s setting time and poured in type IV dental stone (Pars Dandan, Tehran, Iran). All the casts were scanned (Smart Optic, GmbH, Germany), and the resulting STL files were introduced to Geomagic Control X software (3D Systems, USA) (Figure 3). After making virtual cross-sectional cuts along the first premolar cusps’ reference points, the distance between the right and left first premolar buccal and palatal cusps, as well as their inter-fossa distances, were measured with an accuracy of 0.01 mm on both the original model and the study casts (Figure 4). Also, using a global align module, the scan of casts were superimposed on the model scan, using a mean difference of 2000 random points on the model and the casts with an accuracy of 0.001 mm. Data were analyzed using two-way ANOVA and Bonferroni test at the 0.05 significance level.

## Results

According to the results of two-way ANOVA, there was no significant difference between the accuracy of casts made using custom trays with 2-mm spacer thickness and poured at 1-hour and 24-hour intervals after impression removal in terms of the inter-palatal cusp and inter-fossa distances (P=0.48 and 0.83, respectively). However, there was a significant difference in the inter-buccal cusp distance between the accuracy of casts poured 1 hour and 24 hours after impression making (P=0.037).

Furthermore, there was no significant difference between the accuracy of casts made using custom trays with 4-mm spacer thickness and poured at 1 hour and 24 hours after impression removal in terms of inter-buccal/palatal cusp and inter-fossa distances (P>0.05). Also, the mean inter-buccal/palatal cusp and inter-fossa distances on all the casts were <0.5 mm greater than that on the original model.

## Discussion

This study explored a digital technique by using computer-aided design and fused deposition modeling (FDM) technologies for the production of custom trays for the partially edentulous maxilla. The CAD process used for designing the custom trays was Geomagic software, which was applied for the analysis and processing of point-cloud data. In this study, two space settings of 2 and 4 mm were designed between the custom tray and the partially edentulous jaw model as the required space for the impression material. In this order, the tray’s internal surface was designed by 2 or 4 mm, moving from the entire surface data acquired from the primary scan using the offset function of the Geomagic software.^16^

In this study, a 3D FDM printer was used to manufacture custom trays made of polylactic acid (PLA) filament.^16,18,19^ The advantage of digital fabrication of trays is a minimal reserved space deviation as compared with the hand-made trays, which results in higher accuracy and reproducibility of the impression. This is due to eliminating the factor of wax spacer deformation present in hand-made trays.^16^ Also, the time needed to execute the tray fabrication is less than that for the hand-made procedure.

On the other hand, some factors affect the accuracy of the FDM-forming process, including the accuracy of the CAD process and the filament material, the nozzle width, the nozzle, and forming a chamber temperature, layer thickness, and direction.^18,20^ In this study, the nozzle diameter of the 3D FDM printer was 0.4 mm; the accuracy of its XY axis positioning was 10 µm, and the accuracy of its Z-axis positioning was 5 µm. Also, a medium accuracy (200 µm) was chosen.^16^ In order to create a uniform 3D space inside the tray, the tissue surface configuration of the digitally designed tray was fitted with the 3D landmarks of the model. Also, to determine the exact orientation of the tray on the model and uniform distribution of the impression material within the tray, three tissue stops were designed.

The one-step technique using monophasic polyvinyl siloxane is a simple technique that should only be used with custom trays.^5,6,21^ One of the findings in the present study was a small (<1 mm) increase in the inter-abutment distances. This finding might be due to the expansion of the dental stone. This result is different from the findings of Brosky et al^21^ and Tjan et al.^22^ On the other hand, statistical analysis showed no significant differences between the two tray spaces, consistent with the results of Tjan et al^22^ and Caputi & Varvara.^23^ Even increasing the tray space from 2 to 4 mm did not increase the material shrinkage. This finding is consistent with studies that found no differences between varying amounts of tray spaces (1 to 5 mm).^2,5,7-11^ However, the only statistically significant inter-buccal distance was related to the 2-mm spacer group, which showed a higher amount as compared with the original model. Polymerization shrinkage of the impression material toward the largest bulk in the center and also toward the tray walls due to the restriction caused by the impression adhesive during polymerization might be responsible for this finding.

There were no significant differences in the dimensions of the stone models poured at different times, which is due to using additional silicone impression material.^24-26^ However, when a 2-mm spacer was used for trays, the casts poured after the first hour exhibited higher dimensional similarity with the original model with a minimal difference in their means.

## Conclusion

Within the limitations of this study, the following conclusions can be drawn:

1. There was no significant difference between the accuracy of impressions made using trays with 2- and 4-mm spacers.

2. There was no significant difference between the two pouring times (one hour vs. 24 hours) except for the trays with a 2-mm spacer.

## Authors’Contributions

ST and MS contributed to the idea of the research. MJR and PP carried out the experiments. EB and RG collected the data, drafted the manuscript, and finalized the manuscript. All authors have read and approved the final manuscript.

## Acknowledgments

None.

## Funding

None.

## Competing Interests

None.

## Ethics Approval

None.
